# Making a Practice

**Published:** 1924-09

**Authors:** 


### MAKING A PRACTICE.

By a Doctor's Wife.

When my husband was qualified some fifteen years ago, and the money to buy an established practice was not forthcoming, there was nothing for it but to find a house in a likely neighbourhood and wait for patients. We anxiously awaited the arrival of the first patient, and refused to be downcast when a successful local colleague, who had started in the same way, told us that it was three months before anyone came to him for treatment, and then it was a workman who had cut himself while erecting the red lamp outside the surgery!

However, before a week had passed, during a fearful gale, a prolonged peal at the bell, accompanied by a promising cough on the doorstep gladdened our hearts, and an elderly woman was ushered into the consulting-room. Perhaps it would be nearer the mark to say that she floated in, for water poured from the rim of her boat-shaped hat, and she refused to be parted from a streaming umbrella. The consulting-room door closed upon the elated doctor and the bronchitic lady, to reopen rather sooner than I expected, followed by the hurried exit of the patient. My husband returned to tell me that she had given him a long description of her obvious symptoms, and he was about to prescribe for her, when she peered into his face, covering him with moisture from her boat-shaped hat, and said, " Oh ! Ain't you Doctor So-and-so ? Well, I've come to the wrong 'ouse ! " And gathering up her umbrella she departed in great haste.

The pools of water and trail of wet and mud took some time to clear up, and we had not recovered from our amusement before the bell again pealed. It was an urgent call this time, and my husband seized a new hat and bag, and dashed off on his bicycle, only to return the next moment, the wind having blown away his hat and put out his light. Nothing daunted, he set out again, and from that time there was no looking back. But the life of a doctor's wife in a busy practice is full of interest and amusing incidents.

Our house was situated on a busy main road, where a surprising number of accidents occurred ; and one evening a tramcar at a standstill, and an enormous crowd gathering around, proclaimed that yet another had happened. This time the victim was a small child who had dashed in front of a tramcar and was pinned underneath. Her cries could be heard, but nothing could be done until help was sent from the tramway depôt, and the car raised to release her. Eventually she was carried into our house unconscious, badly marked all over the body, and with the base of her skull fractured. She made a wonderful recovery, however, and in a week's time seemed little the worse for her adventure. Such is the buoyancy of youth !

But with success came lack of freedom and leisure, and I learned to dread the frequent ringing of the night-bell after a long day's work regulated by no kindly trade union. Any dinner party was invariably interrupted by a distant call, and one afternoon just as visitors were arriving to a juvenile party, saw a body of policemen carrying a badly hurt man into the consulting room, where he remained for some hours until he was fit to be moved. During this time the hapless young guests remained with their faces glued to the large nursery window, talking in hushed tones, while I endeavoured to comfort the scared occupants of the car which had been responsible for the accident. Still, in spite of such drawbacks, life was a very cheery business, and the routine of an ordinary existence must be dull by comparison, though certainly more restful from a wife's point of view.

### THE ALFRED OF MELBOURNE.

A remarkable story of hospital development is seen in the change from the quietly conducted Alfred Hospital of Melbourne, in 1900, to the huge institution of the present day. Less than twenty-five years ago, so the architect of this hospital, Mr. K. A. Henderson, tells us, it consisted of but three pavilions, which, together with various accessory departments, were surrounded by rookeries and Moreton Bay fig trees. In fact, there were trees everywhere and even a large vegetable garden. Some of the land now occupied by the busiest parts of the hospital was in those days almost unknown country. The first lines of development certainly took the usual direction, namely, the building of new night-nurses' quarters, of further operating theatres and an X-ray room. But the continuous rapidity with which everything was carried out must have been very remarkable. One thing after another was modernised —laundries, boiler-houses, kitchens and stores. The result is magnificent, and Melbourne is to be congratulated upon this part of its medical equipment.

